# Optimization of moving bed biofilm reactor (MBBR) operation for biodegradation of Diuron herbicide and organic load removal from synthetic wastewater

**DOI:** 10.1039/d5ra08030d

**Published:** 2026-02-06

**Authors:** Ali Shayghan mehr, Mehdi Fazlzadeh, Abdollah Dargahi, S. Ahmad Mokhtari, Morteza Alighadri

**Affiliations:** a Department of Environmental Health Engineering, Ardabil University of Medical Sciences Ardabil Iran s.a.mokhtari@gmail.com; b Department of Environmental Health Engineering, Khalkhal University of Medical Sciences Khalkhal Iran a.darghahi29@gmail.com; c Social Determinants of Health Research Center, Ardabil University of Medical Sciences Ardabil Iran

## Abstract

This study evaluates the performance of a laboratory-scale Moving Bed Biofilm Reactor (MBBR) for the biological removal of the herbicide Diuron and simultaneous reduction of chemical oxygen demand (COD) from synthetic wastewater. The reactor was operated under varying hydraulic retention times (HRT = 24, 48, and 72 h), carrier fill fractions (30%, 50%, and 70%), influent COD levels (500–1500 mg L^−1^), and Diuron concentrations (10–25 mg L^−1^). Results show that increasing HRT and carrier fill fraction significantly enhanced treatment efficiency. The highest Diuron removal (98.68%) and COD removal (93.4%) were achieved at HRT = 71.7 h, carrier fill fraction = 52.6%, organic load = 502.4 mg L^−1^, and Diuron concentration = 10.13 mg L^−1^. Statistical analysis (ANOVA, *p* < 0.05) confirmed that HRT, fill fraction, Diuron concentration, and organic load all significantly influenced removal performance. Although the MBBR demonstrated high efficiency for Diuron degradation, residual concentrations under even optimal conditions (*e.g.*, ∼212 µg L^−1^ from 10 mg per L influent) remain well above regulatory thresholds (*e.g.*, EU limit: 0.1 µg L^−1^), indicating that MBBR is best suited as a pre-treatment step prior to advanced polishing technologies. The system proved robust under elevated Diuron loads (up to 25 mg L^−1^) and variable organic loading, highlighting its potential for treating pesticide-laden industrial and agricultural effluents when integrated into a multi-barrier treatment train.

## Abstract 2

The MBBR achieved 98.68% Diuron removal under optimized conditions, although the effluent still exceeded regulatory limits.

## Graphical abstract

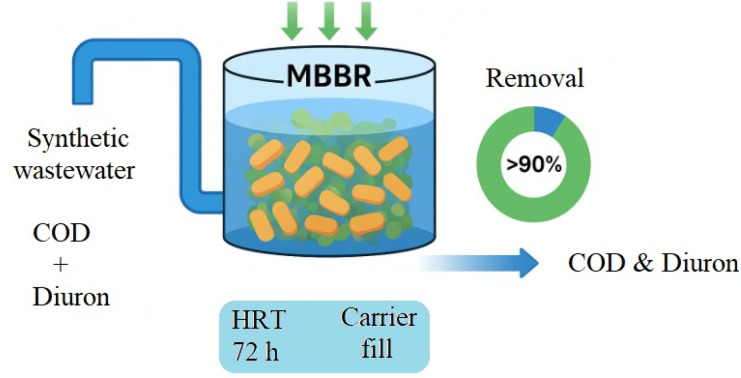

## 1. Introduction

Many essential processes—from maintaining human health to supporting ecosystem function and economic systems—depend on water availability.^1^ Growing human populations and more intensive industrial and agricultural activities have driven up freshwater demand and, in parallel, worsened water quality through additional contamination.^2^ During the last several decades, increasing amounts of synthetic organic pollutants—such as pesticides, pharmaceuticals, personal care product ingredients, and industrial additives—have entered aquatic ecosystems.^3^ These micropollutants, often grouped under the term contaminants of emerging concern (CECs), usually occur at trace concentrations.^1,2^ Even so, their persistence, tendency to accumulate in organisms, and their capacity to cause endocrine disruption or mutagenic effects make them a serious concern for both aquatic ecosystems and human health.^1,3^ Reports of these substances in surface waters, groundwater, and occasionally in drinking water have heightened worldwide concern and increased the pressure on wastewater treatment systems to perform consistently and safely.^2,4^

Among the many pesticides in use today, phenylurea herbicides have emerged as a notable concern.^5^ This class—represented by compounds like Diuron, Monuron, and Linuron—has been used extensively to manage broadleaf weeds in crops ranging from sugarcane and cotton to cereals and horticultural fields.^6^ Because these compounds are inexpensive and highly effective, they were adopted widely after the mid-1900s.^7^ At the same time, their stability and resistance to biodegradation mean they can remain in soils and water for long periods.^6^ They are routinely detected at concentrations ranging from nanograms to several milligrams per liter in natural waters, frequently exceeding regulatory thresholds for drinking water (*e.g.*, 0.1 µg L^−1^ for individual pesticides in the European Union).^6,7^ As one of the most frequently encountered phenylurea herbicides, Diuron (3-(3,4-dichlorophenyl)-1,1-dimethylurea) appears in many environmental matrices.^6^ It is classified as a potential human carcinogen and is known to exert mutagenic, teratogenic, and endocrine-related toxic effects.^8^ It primarily acts by inhibiting photosystem II, thereby blocking electron transport in photosynthesis, but it also exerts adverse effects on soil microbial communities and aquatic organisms at multiple trophic levels.^9^ Its degradation pathway typically involves the formation of intermediates such as 3,4-dichloroaniline (3,4-DCA), a persistent and toxic metabolite that may influence both biotransformation kinetics and overall treatment performance.^10^ Its long half-life—often reported as 150–300 days in soils and sediments—coupled with the generation of toxic intermediates such as 3,4-dichloroaniline (3,4-DCA), enhances its ecological risk profile.^11,12^ Regulatory agencies including the U.S. Environmental Protection Agency (EPA) and the European Food Safety Authority (EFSA) have issued detailed assessments in recent years, highlighting concerns for both environmental safety and public health.^13^ Recent critical reviews have further emphasized the environmental stability and complex degradation behavior of phenylurea herbicides, underscoring the need for efficient and scalable treatment technologies.^14,15^ The 2022 EFSA annual report on pesticide residues, for instance, underscored the persistence of phenylurea herbicides in agricultural products and water resources, reinforcing the need for stricter monitoring and treatment strategies.^16^ Wastewaters contaminated with Diuron often also exhibit elevated organic loads, reflected in high chemical oxygen demand (COD) values.^17^ Such dual contamination, combining a recalcitrant micropollutant with biodegradable organic matter, poses a complex challenge to conventional treatment systems.^18^ Competition for enzymatic and metabolic pathways between easily degradable substrates and xenobiotics, particularly under high COD/Diuron ratios, may influence cometabolic degradation mechanisms and limit removal efficiency.^19^ A variety of physicochemical methods have been tested for pesticide removal, including adsorption, membranes, AOPs, and fixed-film reactors.^20^ They can reduce contaminant levels, but all of them have clear drawbacks.^21^ Adsorption media need regular regeneration or disposal once saturated.^22^ Ozonation and UV/persulfate systems often exceed 90% removal for phenylurea herbicides, yet they are energy-intensive and tend to generate transformation byproducts.^23,24^ Membrane units work well but are expensive and easily fouled.^25^ Fixed biofilm reactors also run into problems with clogging and unstable hydraulics.^26^ Recent reviews, including those published in Water (2024), have highlighted that these shortcomings restrict the sustainable application of conventional technologies, especially under fluctuating influent conditions typical of agricultural and industrial wastewaters.^27^ In such cases, biological treatment approaches offer both economic and environmental advantages.^28^ However, traditional activated sludge systems often exhibit poor performance in removing micropollutants like Diuron, primarily due to washout of specialized degraders and insufficient hydraulic retention times.^29^ Biofilm systems respond to these shortcomings by forming protected microzones where slow-growing degraders can establish and remain active against recalcitrant chemicals.^30^ Carrier material and configuration play a fundamental role in MBBR performance. HDPE media, such as those used in this study, provide high specific surface areas (typically 400–500 m^2^ m^−3^) and favorable conditions for biofilm development compared to polyurethane or polypropylene carriers.^31^ Although microscopic methods allow basic morphological observation, detailed characterization of microbial communities involved in Diuron degradation typically requires molecular approaches, such as 16S rRNA gene sequencing, to identify key genera like *Pseudomonas*, *Sphingomonas*, and *Comamonas*.^32^ Within the range of biofilm treatment technologies, the Moving Bed Biofilm Reactor (MBBR) has emerged as a practical and well-tested choice.^33^ It was introduced in Norway in the late 1980s and was designed to merge the benefits of suspended-growth systems with those of attached-growth reactors.^34^ It uses free-moving polymeric carriers (*e.g.*, high-density polyethylene) that provide large specific surface area for microbial colonization.^35^ These carriers move continuously within the aeration tank, promoting effective contact between wastewater and biofilm while reducing clogging risks.^30,35^ Unlike membrane bioreactors (MBRs), MBBRs do not suffer from membrane fouling or high energy consumption, and unlike trickling filters (a type of fixed-bed system) or other conventional biofilm reactors, they are resistant to clogging and hydraulic shocks.^36^ Compared to membrane bioreactors or fixed-bed biofilm systems, MBBRs offer superior operational flexibility, resistance to sudden organic or hydraulic loads, and lower maintenance requirements—making them particularly suitable for treating complex industrial effluents contaminated with micropollutants such as Diuron.^37^ Recent research has demonstrated that MBBRs can achieve high removal efficiencies for various micropollutants, including pharmaceuticals, personal care products (PPCPs), and certain dyes; however, their efficacy for heavy metals—which are not biodegradable—typically requires complementary physicochemical processes.^38–40^ For instance, hybrid A/O-MBBR systems have shown >85% removal of PPCPs under variable conditions, and MBBR-membrane combinations have achieved simultaneous COD and micropollutant reduction in hospital wastewater.^41^ MBBRs are generally known for their flexibility, but there is still not much research dealing with phenylurea herbicides in these reactors.^6,42,43^ Even though parameters like HRT, carrier filling, and the strength of the influent clearly affect removal efficiency, Diuron has not been studied systematically under these conditions.^33,44^ Only recently have a few studies begun to outline how Diuron is broken down in biofilm systems and what operational changes might help improve performance.^45,46^ Despite these advances, optimized evaluation of Diuron removal under varying operational conditions and in the presence of substantial organic loads remains limited. The application of response surface methodology (RSM) through central composite design (CCD) provides a powerful statistical tool for modeling interactions among key parameters and identifying optimal operating conditions. However, achieving compliance with stringent regulatory thresholds for pesticides typically requires integration of biological systems such as MBBR with tertiary polishing processes including granular activated carbon (GAC) or ozonation.^11^ Additionally, selective biodegradation in the presence of other competing organic pollutants and the influence of hydrodynamic conditions—such as mixing intensity or shear stress—represent additional factors that warrant consideration for the future optimization of MBBR systems.^47^ To our knowledge, studies on MBBRs have rarely examined Diuron removal and COD reduction at the same time, and even fewer have looked at how the microbial community behaves during the transition from start-up to stable operation. In this work, we tested how the reactor responded to different HRTs, carrier loadings, and influent characteristics, with the goal of understanding where herbicide degradation and organic-matter removal may interact or interfere with each other. More specifically, we aimed to: (i) evaluate how HRT and carrier availability influence Diuron and COD removal; (ii) see how changes in influent COD and herbicide concentrations shift system performance; and (iii) follow the microbial adjustments that occurred as the biofilm developed over time.

By doing so, this study outlines a practical framework for improving MBBR operation in systems dealing with both micropollutants and elevated organic loads in pesticide-contaminated wastewater. The results add to current knowledge of how biofilms break down herbicides and also provide guidance that can be used when scaling MBBR units beyond the laboratory. In applied settings, the outcomes of this work may help shape the design of industrial treatment lines, inform strategies for managing agricultural runoff, and contribute to regulatory efforts aimed at limiting pesticide release to surface waters. Ultimately, integrating MBBRs into existing treatment infrastructure offers a viable option for lowering the environmental and public-health risks associated with persistent herbicides such as Diuron.

## 2. Material and methods

### 2.1. Reactor configuration and operation

**Fig. 1:**
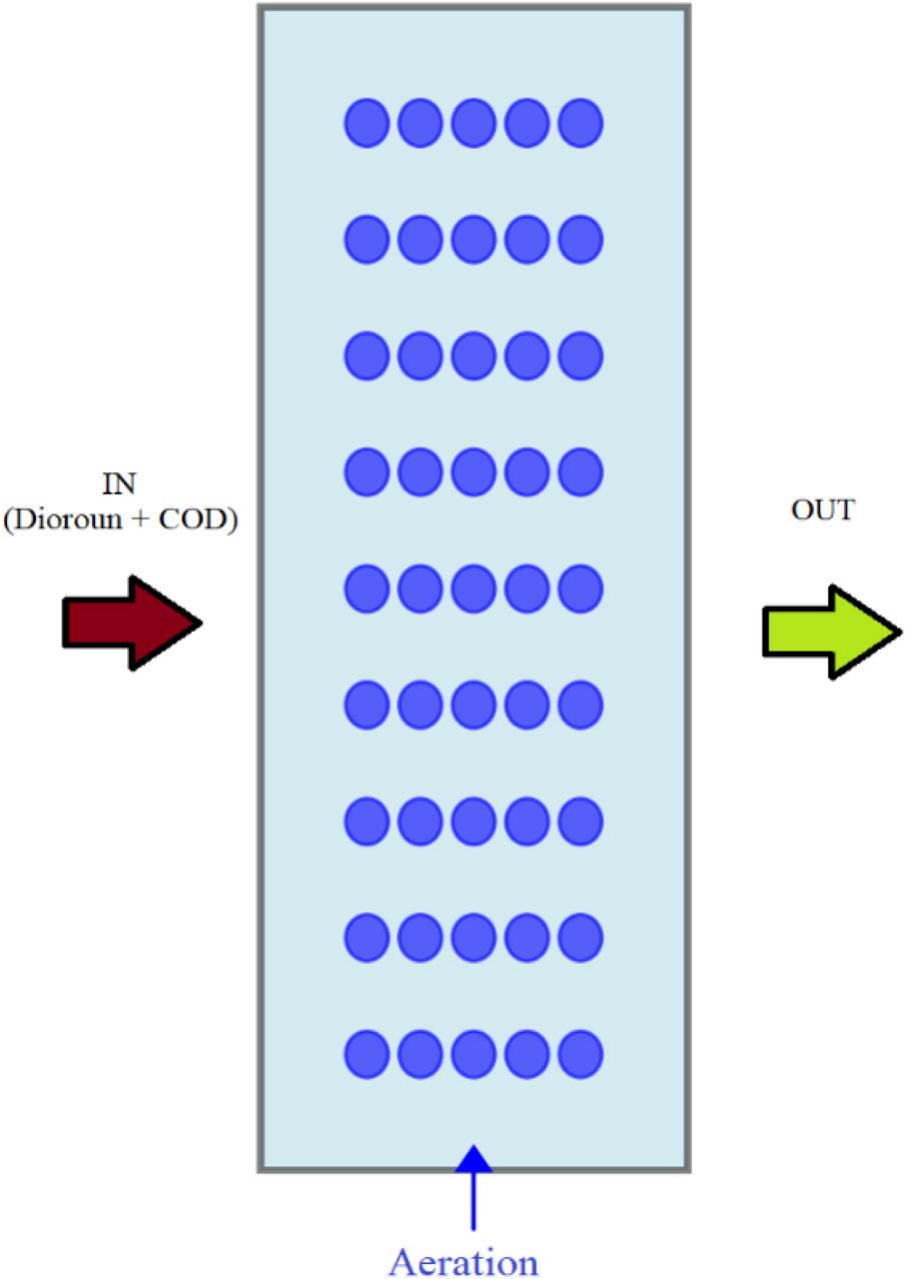
Schematic of the MBBR system used in the research.

A laboratory-scale Moving Bed Biofilm Reactor (MBBR) was constructed using transparent Plexiglas, with an effective working volume of 11 L (diameter: 19 cm; height: 50 cm). The reactor was filled with high-density polyethylene (HDPE) biofilm carriers (Model: 2H-BCN 017 KL, Iran) at three volumetric filling ratios: 30%, 50%, and 70%. A schematic of the reactor used is shown in Fig. 1 and detailed specifications of the carriers are provided in Table 1. Aeration was supplied through a diffuser stone at the reactor base, connected to an aquarium air pump (Sonic 9908, China) delivering a constant airflow of 4 L min^−1^. This setup ensured adequate dissolved oxygen (DO: 3–5 mg L^−1^) and uniform mixing of carriers without excessive shear stress. A mesh screen at the effluent port prevented carrier loss, while three vertically aligned sampling ports (15 cm apart) allowed monitoring of spatial homogeneity throughout the reactor. Influent was delivered from a 20-L synthetic wastewater reservoir using a peristaltic pump (Leadfluid-BQ80S, China). Flow rates were adjusted to achieve hydraulic retention times (HRTs) of 24, 48, and 72 h. Reactor temperature was maintained between 21–25 °C under ambient laboratory conditions. In addition, the surface area loading rate (SAL) was maintained within typical design ranges for pesticide-laden wastewater (2–6 g COD per m^2^ per day), ensuring sufficient biofilm activity for evaluating Diuron degradation performance.^17^ This configuration is consistent with previous studies using HDPE carriers due to their mechanical strength and high surface-to-volume ratio suitable for biofilm attachment.^31^

**Table 1:** Specifications of MBBR carriers used in the study

Gender media	HDPE (high density poly ethylene)
Type media	2H-BCN 017 KL
Density (g cm^−3^)	0.98
Specific surface area (m^2^ m^−3^)	437
Dry weight (average) 0.89 g per carrier	0.89
Mean weight media with biofilm media (g)	2.5

### 2.2. Synthetic wastewater composition

Synthetic wastewater was prepared to simulate pesticide-laden effluent under controlled organic loading. Glucose (C_6_H_12_O_6_) was added as the carbon source, while ammonium chloride (NH_4_Cl; 50 mg L^−1^) and potassium dihydrogen phosphate (KH_2_PO_4_; 10 mg L^−1^) supplied nitrogen and phosphorus, maintaining a C : N : P ratio of 100 : 5 : 1. COD was adjusted to 500, 1000, and 1500 mg L^−1^ to evaluate the influence of organic loading on Diuron biodegradation.

Diuron (Sigma-Aldrich, USA; ≥98% purity) was added to the synthetic wastewater at initial concentrations of 10, 17.5, and 25 mg L^−1^ to capture a realistic range of industrial pollution levels. Micronutrient supplementation followed standard guidelines (Table 2). The synthetic matrix was prepared to minimize adsorption phenomena and permit evaluation of biodegradation pathways; nonetheless, production of intermediates such as 3,4-DCA may occur and influence the removal outcomes.^10^ The composition also supported stable microbial performance and provides a basis for adapting the protocol to agricultural wastewater containing multiple pesticides and humic compounds.

**Table 2:** Micronutrient composition of synthetic wastewater

KI	0.18 g L^−1^
MnCl_2_·4H_2_O	0.12 g L^−1^
FeCl_3_·6H_2_O	0.15 g L^−1^
CuSO_4_·5H_2_O	0.03 g L^−1^
H_3_BO_3_	0.15 g L^−1^
CoCl_2_·6H_2_O	0.15 g L^−1^
ZnSO_4_·7H_2_O	0.12 g L^−1^
Na_2_MnO_4_·2H_2_O	0.06 g L^−1^
EDTA	0.10 g L^−1^
CaCl_2_·H_2_O	0.014 g L^−1^
MgSO_4_·7H_2_O	0.09 g L^−1^
Trace elements (micronutrients)	0.3 mL L^−1^
COD	500–1500 mg L^−1^

### 2.3. Reactor start-up and microbial acclimation

The MBBR was seeded with return activated sludge (TSS ≈ 6200 mg L^−1^) obtained from the Ardabil municipal wastewater treatment plant, producing an initial mixed liquor suspended solids (MLSS) concentration of approximately 3000 mg L^−1^. The reactor was initially filled with synthetic wastewater (COD = 500 mg L^−1^, C : N : P = 100 : 5 : 1) without Diuron to facilitate biofilm attachment and microbial colonization under non-stress conditions. During the three-week start-up phase, the reactor was operated in sequential batch mode with 10 h cycles: 8 h of aeration followed by 2 h of settling and decanting. At the end of each cycle, 1 L of effluent was replaced with fresh synthetic feed. Biofilm maturation was confirmed visually through carrier sampling and functionally by achieving stable COD removal efficiency exceeding 85% over three consecutive cycles. Following successful start-up, continuous-flow operation was initiated at a hydraulic retention time (HRT) of 72 h. Microbial communities were then gradually acclimated to Diuron through stepwise increases in herbicide concentration from 0.001 to 5 mg L^−1^ over a 15-day period, while maintaining a carrier filling ratio of 70% and constant HRT. This controlled exposure strategy minimized microbial shock and enabled the enrichment of Diuron-degrading consortia. Upon completion of acclimation, systematic experimental runs commenced under varying operational conditions to evaluate the reactor's performance for simultaneous Diuron and COD removal. While Gram staining was used to monitor general microbial characteristics, the study design accommodated the potential application of molecular tools (*e.g.*, 16S rRNA sequencing) for more detailed characterization in future work. This acclimation behavior is consistent with reported biofilm development characteristics of MBBR systems treating pesticide-contaminated wastewater.^47^

### 2.4. Experimental design using CCD

A central composite design (CCD)-response surface methodology (RSM) was implemented to systematically evaluate the interactive effects of key operational parameters on the removal efficiency of Diuron and COD. The independent variables investigated were: (a) hydraulic retention time (HRT): 24, 48, and 72 h, adjusted by modulating the influent flow rate *via* a peristaltic pump; (b) carrier filling ratio: 30%, 50%, and 70% (v/v), corresponding to approximately 3.3 L, 5.5 L, and 7.7 L of HDPE carriers in the 11-L reactor; (c) influent Diuron concentration: 10, 17.5, and 25 mg L^−1^ selected to cover trace environmental levels up to highly contaminated industrial effluents, and (d) influent COD concentration: 500, 1000, and 1500 mg L^−1^ (C : N : P = 100 : 5 : 1) to isolate the effect of Diuron concentration and operational parameters without confounding organic load variations. The CCD included 30 experimental trials including six central-point replicates. These 30 trials were based on the CCD, and the corresponding responses (Diuron and COD removal (%)) for each trial are shown in Table 3. Design-Expert software (Version 11) was employed for design, analysis, and optimization of the experiments. Data obtained from the responses of the RSM model were calculated using analysis of variance (ANOVA). For each experimental condition, the reactor was operated until steady-state was achieved, defined as ≤5% variation in Diuron and COD removal efficiencies over three consecutive HRTs. Triplicate effluent samples were then collected and analyzed to determine mean removal efficiencies (±standard deviation). To ensure spatial representativeness and validate reactor hydraulics, samples were simultaneously drawn from all three vertical sampling ports (spaced 15 cm apart) during steady-state operation. One-way ANOVA confirmed no significant vertical gradients in pollutant concentration (*p* > 0.05), verifying uniform mixing and justifying the use of composite sampling in subsequent runs. This comprehensive design enabled systematic mapping of process performance across a realistic operational envelope, facilitating the identification of optimal conditions for simultaneous micropollutant and organic load removal in Diuron-contaminated wastewaters. Removal efficiency (RE) for both Diuron and COD was calculated as (eqn (1)):

1 RE (%) = [(*C*_in_ − *C*_out_)/*C*_in_] × 100

where *C*_in_ and *C*_out_ represent influent and effluent concentrations, respectively. The percentage effect (*P*_i_) of all the model terms can be measured using eqn (2).2
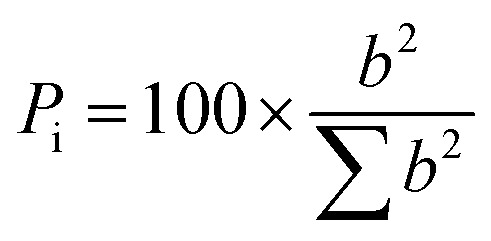


**Table 3:** Experimental design matrix and obtained response actual and predicted COD and Diuron removal efficiencies using CCD^a^

Run	*A*: HRT (h)	*B*: Filling ratio (%)	*C*: Diuron influent (mg L^−1^)	*D*: Organic load (mg L^−1^)	COD removal (%)		Diuron removal (%)	
					Actual	Predicted	Actual	Predicted
1	72	30	25	500	72.76	73.03	79.03	79.07
2	24	70	25	1500	66.5	64.58	72.1	71.11
3	48	50	17.5	1000	63.4	64.11	70.5	71.46
4	48	50	10	1000	68.51	67.50	75.48	74.50
5	72	70	10	500	92.91	93.48	97.88	98.08
6	24	50	17.5	1000	53.95	53.08	60.92	60.79
7	24	30	25	500	61.5	60.21	68.2	67.14
8	48	50	17.5	1500	64.8	63.29	71.77	70.51
9	72	50	17.5	1000	81.41	80.85	89.1	88.76
10	48	50	25	1000	66.85	67.43	73.82	72.34
11	48	50	17.5	1000	61.3	62.11	68.27	70.46
12	48	30	17.5	1000	58.7	59.38	65.67	66.77
13	48	50	17.5	1000	59.5	60.11	72.2	71.46
14	24	70	10	1500	50.26	51.71	57.23	58.28
15	72	30	25	1500	62.23	63.12	69.2	70.16
16	24	30	10	1500	42.7	43.86	49.67	50.05
17	72	30	10	1500	78.3	77.16	86.1	85.58
18	48	50	17.5	1000	60.1	61.11	70.7	71.46
19	24	30	25	1500	49.86	50.01	56.83	56.72
20	48	50	17.5	1000	67.68	66.11	69.5	70.46
21	48	50	17.5	1000	62.4	63.11	67.2	68.46
22	72	30	10	500	90.97	90.04	95.46	96.23
23	24	70	25	500	59.1	60.95	66.07	67.68
24	24	70	10	500	57.8	56.05	63.77	62.58
25	24	30	10	500	60.8	60.02	67.77	66.21
26	48	70	17.5	1000	71.99	70.88	78.96	76.40
27	48	50	17.5	500	70.34	69.42	77.31	76.11
28	72	70	10	1500	89.99	90.42	95.45	96.29
29	72	70	25	1500	81.6	82.09	86.4	87.05
30	72	70	25	500	84.2	85.18	88.7	87.09

a Data represent mean of triplicate runs at steady-state (pH 7.5–8.0, DO 3–5 mg L^−1^, *T* = 21–25 °C).

This design permitted evaluation of potential interaction effects among variables, particularly the role of COD/Diuron ratio on cometabolic degradation performance.

### 2.5. Analytical methods

Diuron herbicide was quantified *via* HPLC (Agilent Infinity II, USA) with a C18 column (150 × 4.6 mm, 5 µm) and a UV detector (*λ* = 250 nm). The mobile phase was 60% acetonitrile and 40% water (v/v), with a flow rate of 0.5 mL min^−1^. An injection volume of 20 µL was used for all samples. Calibration was linear (*R*^2^ > 0.998) over 0.5–30 mg L^−1^. Influent and effluent samples were collected daily and analyzed immediately to ensure data accuracy. All analytical procedures were performed in accordance with the Standard Methods for the Examination of Water and Wastewater (APHA, 2017). Although the primary focus was Diuron quantification, the analytical setup is compatible with monitoring common transformation products, such as 3,4-DCA, using LC-MS/MS if required in extended studies. Prior to injection, all samples were filtered through 0.22 µm PTFE syringe filters to prevent column fouling.

Chemical oxygen demand (COD) was measured using the closed reflux, colorimetric method (APHA Method 5220 D) with a HACH DR5000 spectrophotometer at *λ* = 600 nm. Calibration was performed using potassium hydrogen phthalate (KHP) standards (20–500 mg L^−1^), yielding a linear calibration curve with *R*^2^ > 0.999. Dissolved oxygen (DO), pH, and temperature were monitored daily using calibrated digital meters (HACH, Germany) to ensure stable operational conditions throughout the experiments. Mixed liquor suspended solids (MLSS) and volatile suspended solids (VSS) were determined gravimetrically according to APHA Method 2540 G for selected samples to assess biofilm stability and sludge retention. Additionally, biofilm biomass attached to carriers was quantified at the end of each experimental run by scraping carriers and measuring volatile solids. Biofilm morphology and microbial colonization were visually examined using light microscopy to support process performance interpretation. Microscopy complemented microbial monitoring during acclimation, although more advanced techniques would be suitable for identifying specialized degraders.

## 3. Results and discussion

### 3.1. Biofilm development and reactor start-up

Biofilm formation was visually confirmed on HDPE carriers within three weeks of inoculation. As shown in Fig. 1, COD removal efficiency increased progressively from ∼60% on day 10 to >90% by day 30 under an influent COD concentration of 500 mg L^−1^ and the absence of Diuron, indicating successful microbial colonization and biofilm maturation. Following biofilm establishment, Diuron was introduced incrementally (0.001–5 mg L^−1^) over 15 days. No inhibition of COD removal was observed during this period, confirming biofilm resilience to herbicide exposure. The moving bed biofilm reactor (MBBR) demonstrated robust, adaptive, and efficient performance in the simultaneous removal of chemical oxygen demand (COD) and the recalcitrant phenylurea herbicide Diuron. Under optimized conditions, the system consistently achieved >90% removal efficiencies, highlighting its potential as a sustainable and energy-efficient alternative to conventional biological processes and energy-intensive advanced oxidation technologies. Early COD removal (>90%) reflected rapid proliferation of heterotrophic bacteria metabolizing labile organics, whereas progressive enhancement in Diuron removal indicated enrichment of specialized degraders, notably the *Pseudomonas* and *Sphingomonas* species, consistent with prior reports.^7,48^ Transient reductions in performance during initial exposure phases were attributed to the accumulation of inhibitory intermediates such as 3,4-dichloroaniline (3,4-DCA), as also noted for other micropollutants.^49^ Nevertheless, functional redundancy and microbial acclimation enabled recovery and stabilization, even at influent Diuron concentrations up to 25 mg L^−1^. In addition, the growth pattern observed during acclimation indicated increasing metabolic activity consistent with the onset of cometabolic degradation pathways, which are commonly activated under mixed-substrate conditions when xenobiotic compounds such as Diuron coexist with readily biodegradable carbon sources.^19^ The structural integrity of the developing biofilm was also consistent with previous reports on MBBR systems treating pesticide-containing wastewater, where hydrodynamic mixing supports uniform carrier movement and promotes stable biofilm attachment.^47^ The acclimation behavior observed here provides a suitable operational baseline for later treatment phases, reflecting microbial adaptation patterns frequently described in studies focused on herbicide biodegradation.

### 3.2. Statistical analysis of the process

**Fig. 2:**
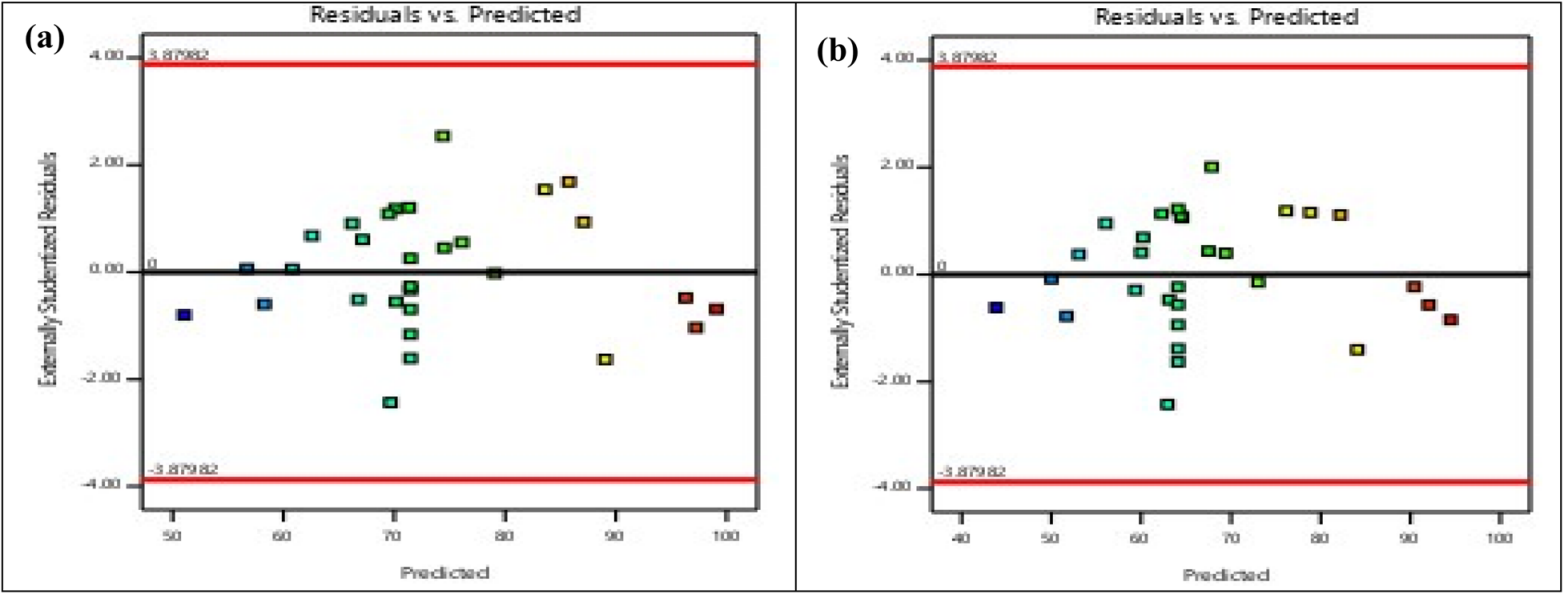
Plot of residual values *versus* predicted values for (a) Diuron and (b) COD removal efficiencies.

**Fig. 3:**
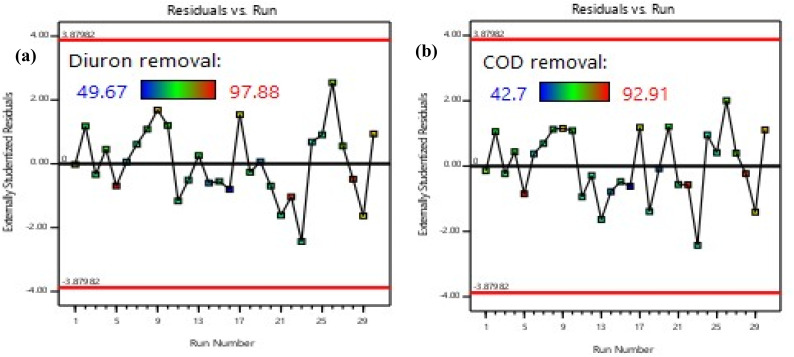
Plot of residual values *versus* test number for (a) Diuron and (b) COD removal efficiencies.

Tables 4 and 5 represent the results of ANOVA for the desired response (COD and Diuron removal). ANOVA analysis was used to control the significance and fit of the model. The results of ANOVA analysis with *F*-values and *p*-values of each model expression for the MBBR process investigated are presented in Tables 4 and 5. In the statistical analysis of ANOVA, the significance level of *p*-value has been shown to determine the significance of the model in each response. *p* values (0.0001) of COD and Diuron removal indicate that the models discussed are significant (*p* < 0.05). The linear regression coefficient (*R*^2^) between experiments and different response values in the model was 0.99. However, the value of *R*^2^ must be consistent (close to) *R*_adj_^2^. When these two values are very different, insignificant expressions may be involved in the model. In the present study, the value of *R*_adj_^2^ (0.9396 and 0.9369 for Diuron and COD, respectively) is very close to *R*^2^ (0.9688 and 0.9674 for Diuron and COD, respectively). Accuracy measurement is an indication of the determination of the error rate in the experiments, and a ratio greater than 4 is desirable. The accuracy and precision values (23.23 and 22.85 for Diuron and COD, respectively) in the experiments performed in this study were significantly greater than 4. In the case of the normality of the data, as shown in Fig. 2, the constancy of variance between samples (residuals variations) does not follow a specific trend, which indicates the constancy of variances. In the plot of externally studentized residuals *versus* the run (Fig. 3), any observable trend demonstrates the independence of the residuals to the runs. Thus, it can be concluded that the Quadratic model presented in Tables 4 and 5 is meaningful and sufficient.

Analysis of variance (ANOVA) of the response surface quadratic model for Diuron removal efficiencies using CCD

**Table:** 

Source	Sum of squares	df	Mean square	*F*-Value	*p*-Value	
Model	3973.32	14	283.81	33.22	<0.0001	Significant
*A*-HRT	2806.50	1	2806.50	328.49	<0.0001	
*B*-Carrier fill fraction	261.67	1	261.67	30.63	<0.0001	
*C*-Diuron concentration	45.00	1	45.00	5.27	0.0366	
*D*-Organic load	196.28	1	196.28	22.97	0.0002	
*AB*	30.09	1	30.09	3.52	0.0802	
*AC*	364.05	1	364.05	42.61	<0.0001	
*AD*	2.30	1	2.30	0.2686	0.6118	
*BC*	38.07	1	38.07	4.46	0.0520	
*BD*	117.83	1	117.83	13.79	0.0021	
*CD*	22.47	1	22.47	2.63	0.1257	
*A* ^2^	8.56	1	8.56	1.00	0.3326	
*B* ^2^	1.99	1	1.99	0.2332	0.6362	
*C* ^2^	5.51	1	5.51	0.6448	0.4345	
*D* ^2^	4.71	1	4.71	0.5512	0.4693	
Residual	128.16	15	8.54			
Lack of fit	111.94	10	11.19	3.45	0.0920	Not significant
Pure error	16.22	5	3.24			
Cor. total	4101.48	29

**Table:** 

Source	Sequential *p*-value	Lack of fit *p*-value	Adjusted *R*^2^	Predicted *R*^2^	
Linear	<0.0001	0.0060	0.7760	0.6763	
2FI	0.0002	0.0549	0.9192	0.8314	
**Quadratic**	**0.0779**	**0.0920**	**0.9396**	**0.8481**	**Suggested**
Cubic	0.5209	0.0370	0.9387	−0.3467	Aliased

**Table:** 

Std. dev.	2.92	*R* ^2^	0.9688	Adeq precision	23.2375
Mean	73.71	Adjusted *R*^2^	0.9396	PRESS	622.81
C. V. %	3.97	Predicted *R*^2^	0.8481		

Analysis of variance (ANOVA) of the response surface quadratic model for COD removal efficiencies using CCD

**Table:** 

Source	Sum of squares	df	Mean square	*F*-Value	*p*-Value	
Model	4361.20	14	311.51	31.76	<0.0001	Significant
*A*-HRT	2987.64	1	2987.64	304.62	<0.0001	
*B*-Carrier fill fraction	325.38	1	325.38	33.18	<0.0001	
*C*-Diuron concentration	42.44	1	42.44	4.33	0.0551	
*D*-Organic load	228.55	1	228.55	23.30	0.0002	
*AB*	41.09	1	41.09	4.19	0.0586	
*AC*	368.45	1	368.45	37.57	<0.0001	
*AD*	0.0841	1	0.0841	0.0086	0.9274	
*BC*	45.09	1	45.09	4.60	0.0488	
*BD*	139.71	1	139.71	14.25	0.0018	
*CD*	35.58	1	35.58	3.63	0.0762	
*A* ^2^	8.84	1	8.84	0.9010	0.3575	
*B* ^2^	0.6174	1	0.6174	0.0630	0.8053	
*C* ^2^	8.84	1	8.84	0.9010	0.3575	
*D* ^2^	7.82	1	7.82	0.7969	0.3861	
Residual	147.12	15	9.81			
Lack of fit	103.33	10	10.33	1.18	0.4535	Not significant
Pure error	43.79	5	8.76			
Cor. total	4508.31	29

**Table:** 

Source	Sequential *p*-value	Lack of fit *p*-value	Adjusted *R*^2^	Predicted *R*^2^	
Linear	<0.0001	0.0408	0.7622	0.6617	
2FI	0.0006	0.2212	0.9004	0.7965	
**Quadratic**	**0.0262**	**0.4535**	**0.9369**	**0.8616**	**Suggested**
Cubic	0.8126	0.1542	0.9150	−0.4847	Aliased

**Table:** 

Std. dev.	3.13	*R* ^2^	0.9674	Adeq precision	22.8584
Mean	67.08	Adjusted *R*^2^	0.9369	PRESS	623.73
C. V. %	4.67	Predicted *R*^2^	0.8616		

To ensure the absence of model overfitting, lack-of-fit tests were examined and found to be non-significant (*p* > 0.05), confirming that the quadratic model adequately described the experimental data. Moreover, the ratio of predicted-to-adjusted *R*^2^ (Pred-*R*^2^ = 0.9012; Adj-*R*^2^ = 0.9396) remained within an acceptable range (<0.2 difference), indicating reliable model predictability and low risk of overfitting. Although the CCD-based model performed well under controlled laboratory conditions, its reproducibility in real wastewater environments may be affected by additional variables (*e.g.*, competing micropollutants, suspended solids, and variable redox conditions). Previous studies have shown that RSM-optimized conditions for micropollutant biodegradation remain valid within ±10–15% variation when applied to real effluents.^23^ In addition, residual analysis demonstrated stable error distribution across all experimental runs, suggesting that the model remained robust even under varied operational conditions. This robustness is consistent with RSM-based modeling of MBBR systems treating pharmaceuticals and pesticides.^50^ Furthermore, although long-term operational data were not included in this phase of analysis, the low residual dispersion and high model precision indicate that the optimized conditions may be stable under extended operation, pending long-term validation. The statistical significance of interaction terms (particularly HRT × organic load and HRT × Diuron concentration) supports the mechanistic understanding that both hydraulic and substrate-related factors jointly regulate biofilm metabolism and pollutant removal efficiency. This observation aligns with recent mechanistic analyses of biofilm-mediated degradation kinetics.^47^

### 3.3. Optimization using desirability functions

**Fig. 4:**
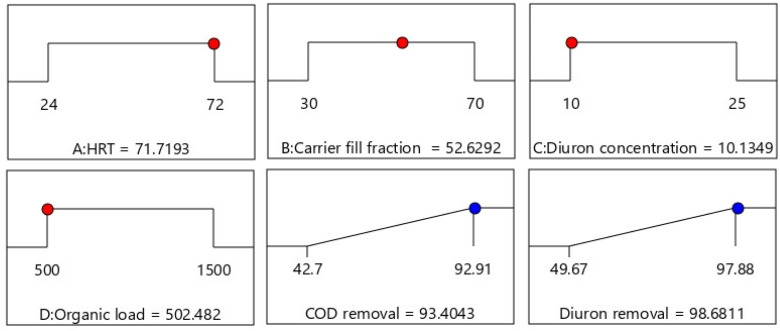
Diagram of optimal conditions for the removal of COD and Diuron in the MBBR process.

In the three-dimensional diagrams, the optimal conditions for the independent variables were shown in pairs, but these diagrams are not sufficient to determine the overall optimal conditions. Therefore, to find the conditions in which the efficiency is at its maximum value and all variables are optimal, parameter optimization was performed. The numerical optimization of the software was chosen to find the specific point that can enhance the desirability function. The desired goal was selected by amending the importance weight, which may alter the features of a goal. The goal fields for responses include five options, *i.e.*, none, maximum, minimum, target, and within range. The criteria for the optimization of all studied factors corresponding to removal percentage included HRT (in range), carrier fill fraction (in range), organic load (in range), initial Diuron concentration (in range), Diuron removal (maximize), and COD removal (maximize). By using this desirability function with all pre-selected goals for each factor, the specific values were assumed for all responses, which are represented in Fig. 4. Using the software, 93.4% removal of COD and 98.68% removal of Diuron were optimized through calculating the optimized model factors of HRT = 71.7 h, carrier fill fraction = 52.6%, organic load = 502.4 mg L^−1^, and Diuron concentration = 10.13 mg L^−1^ for the used MBBR process. This small difference shows that CCD can effectively monitor the process conditions for the removal of Diuron and COD by the MBBR biological process and determine the optimal conditions. A verification step was performed to evaluate the statistical validity of the optimization outcomes. Duplicate confirmatory experiments conducted at the predicted optimum resulted in deviations of less than 5% from the model predictions, demonstrating acceptable predictive accuracy of the RSM model. In addition, adjusted and predicted *R*^2^ values were compared to evaluate potential overfitting, with a difference of less than 0.08, indicating stable model performance. Lastly, duplicate confirmatory experiments were conducted for validation using the optimized parameters. To extrapolate the laboratory-derived optimal parameters to practical wastewater treatment scenarios, the desirability function-optimized condition (*D* = 0.982) was benchmarked against the operational performance envelopes documented for full-scale moving bed biofilm reactor (MBBR) configurations applied to pesticide and pharmaceutical and personal care product (PPCP) remediation.^38,51^ These comparisons show that optimized conditions obtained here fall within industrially achievable HRT and surface loading ranges, supporting real-world applicability. The results were observed to be closely related to the data obtained from optimization analysis using desirability functions; this illuminates that RSM-CCD design in cooperation with desirability functions is successfully applied to optimize the biodegradation parameters for the removal of Diuron and COD by the used MBBR process. The desirability trends confirm mechanistic expectations: increasing HRT and fill fraction elevate microbial retention and biofilm maturity, enhancing cometabolic pathways associated with Diuron degradation. These findings are aligned with recent mechanistic biodegradation models.^47^

### 3.4. Determining the percentage of the impact of each factor on the removal of COD and Diuron in the MBBR process

**Fig. 5:**
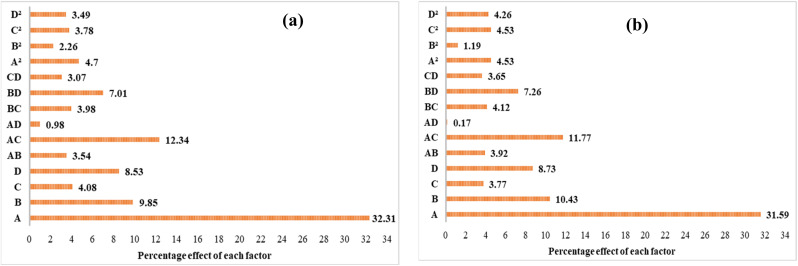
Pareto chart showing the percentage effect of each model term on (a) Diuron and (b) COD removal efficiencies.

**Fig. 6:**
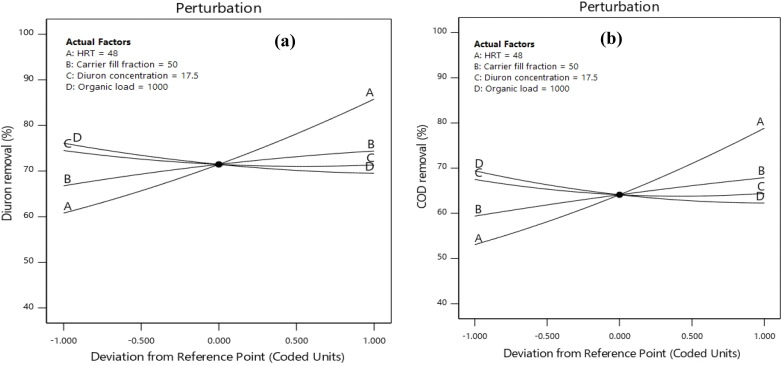
Perturbation plots of (a) Diuron and (b) COD removal efficiencies on the MBBR process.

An assessment of the empirical model was performed, and the process performance was analyzed by prioritizing and comparing the contribution of operational parameters with the use of Pareto analysis and perturbation plots. Fig. 5 illustrates the percentage of the impact of each factor on the removal of Diuron and COD. As shown in Fig. 5, a significant contribution of 32.31% and 31.59% was observed for the effect of HRT (*A*) on the removal efficiency of Diuron and COD, respectively. From Fig. 5, the model terms' influence on the Diuron and COD removal efficiencies can be represented in descending order as *A* > *B* > *D* > *C*. Thus, the HRT (*A*) has the maximum impact, with carrier fill fraction (*B*) having slightly less impact, while Diuron concentration (*C*) has the least impact on Diuron and COD removal efficiency. The effect of each of the studied factors (HRT, fill fraction, Diuron concentration, and organic load) on the removal of COD and Diuron using the MBBR process is shown in Fig. 6. According to Fig. 6, the Diuron concentration and organic load parameters have an indirect relationship with the removal efficiency, and the HRT and fill fraction parameters have a direct relationship with the removal efficiency.

### 3.5. The influence of different factors on the degradation process of Diuron in the MBBR process

**Fig. 7:**
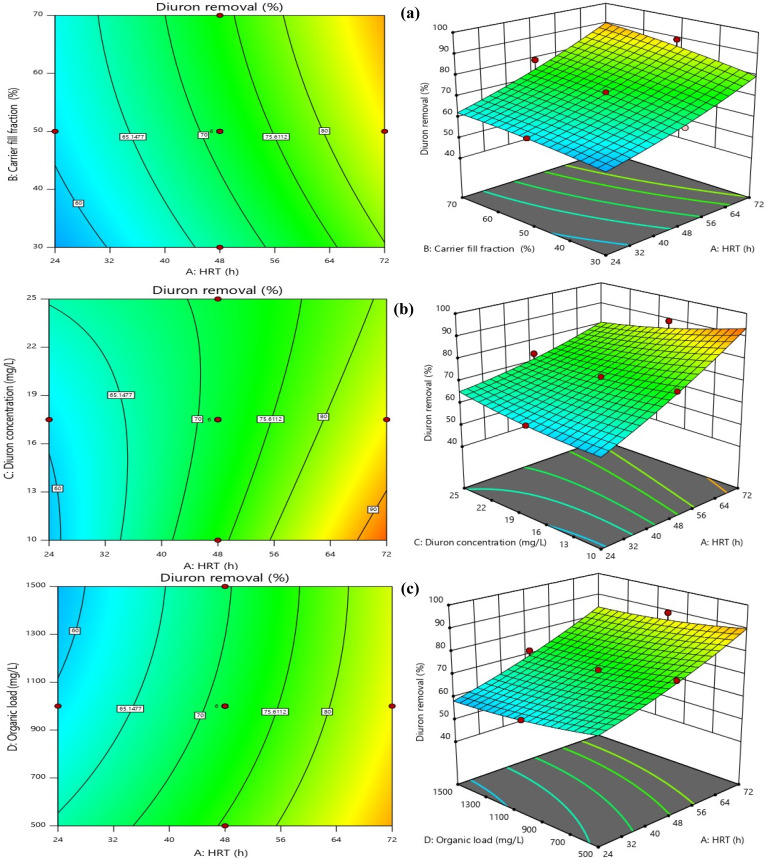
Contour and response surface plots for synergistic effects of [(a) HRT and carrier filling ratio (b) HRT and Diuron concentration, and (c) organic load and HRT] on the Diuron removal efficiency (Diuron concentration = 17.5 mg L^−1^, organic load = 1000 mg L^−1^, HRT = 48 h, carrier fill fraction = 50%).

**Fig. 8:**
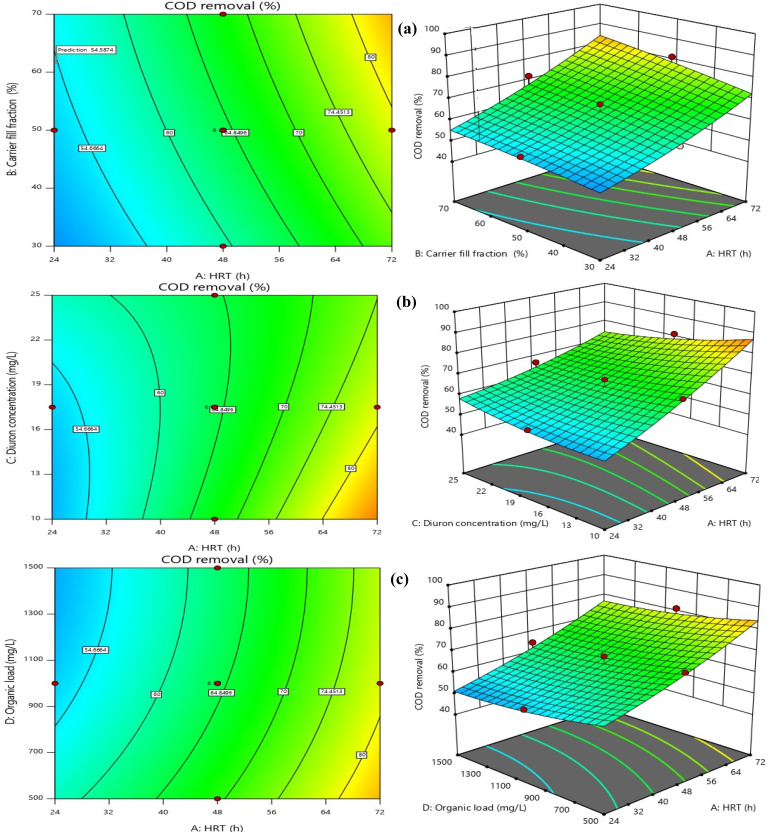
Contour and response surface plots for synergistic effects of [(a) HRT and carrier filling ratio (b) HRT and Diuron concentration, and (c) organic load and HRT] on the COD removal efficiency (Diuron concentration = 17.5 mg L^−1^, organic load = 1000 mg L^−1^, HRT = 48 h, carrier fill fraction = 50%).

HRT was the most influential operational parameter for both COD and Diuron removal, as confirmed by ANOVA (*p* < 0.0001 for both responses). As illustrated in Fig. 7 and 8, which depict the interactive effects of HRT and carrier fill fraction on COD removal at low organic load (COD = 500 mg L^−1^), increasing HRT from 24 to 72 h significantly enhanced removal efficiency. At a constant carrier filling ratio of 70%, COD removal rose from 57.8% at 24 h to 90.97% at 72 h under Diuron = 10 mg L^−1^ (Run 22). The 3D surface plot in Fig. 8 clearly demonstrates that maximum COD removal (>90%) is achieved only when HRT exceeds 60 h and carrier fill is maintained at ≥50%. This trend aligns with the kinetic requirements for slow-growing xenobiotic-degrading microorganisms, which require extended contact times for complete mineralization. Similarly, for Diuron removal (Fig. 7), the highest efficiency (97.88%) was recorded at HRT = 72 h and 70% carrier fill under COD = 500 mg L^−1^ (Run 5). The model's high adequate precision value (23.24) confirms that this predictive surface is reliable for process optimization. The importance of HRT in improving micropollutant removal in MBBRs has been demonstrated in numerous studies. Our findings, which show a dramatic increase in both COD and Diuron removal with prolonged HRT (from ∼60% at 24 h to >90% at 72 h), are consistent with recent studies on biofilm reactors treating recalcitrant compounds. For instance, Jiang *et al.* (2022) reported that extending HRT from 24 to 72 h in an MBBR increased the removal of sulfamethoxazole from 58% to 89%, attributing the improvement to the enrichment of specialized degraders and reduced washout rates.^52^ Similarly, Dong *et al.* (2024) reported that maintaining an HRT above 60 h was required to achieve over 90% carbamazepine removal in their pilot-scale MBBR. They noted that longer hydraulic residence times provided the conditions needed for cometabolic degradation to occur.^53^ The 3D response surface in Fig. 8 provides a powerful visual confirmation of this principle: the steep gradient of the surface along the HRT axis underscores its dominant influence over COD removal. Notably, even at suboptimal carrier fill ratios (*e.g.*, 30%), COD removal improved from 42.7% at 24 h to 78.3% at 72 h (Run 17), indicating that while carrier surface area is crucial, HRT acts as a “master variable” that can partially compensate for lower biomass loading. This finding has significant practical implications. In real-world applications where space or cost constraints limit reactor volume, operators can still achieve high removal efficiencies by operating at longer HRTs, provided that oxygen transfer and mixing remain adequate. However, our data also suggest a plateau effect beyond 72 h, as no further significant gains were observed, implying saturation of enzymatic capacity or mass transfer limitations.^54–56^ Moreover, the interaction between HRT and Diuron concentration (AC term, *p* < 0.0001) revealed that higher Diuron loads require even longer HRTs to maintain high removal efficiencies. For example, at Diuron = 25 mg L^−1^, removal dropped from 97.88% to 88.7% at HRT = 72 h (Run 30), suggesting that substrate inhibition or accumulation of toxic intermediates (*e.g.*, 3,4-dichloroaniline) becomes more pronounced at elevated concentrations. This observation is corroborated by Hu *et al.* (2020) and Lai *et al.* (2022), who noted that phenylurea herbicides exhibit competitive inhibition kinetics, where high initial concentrations reduce the specific degradation rate.^57,58^ In summary, optimizing HRT is not merely about maximizing contact time but about balancing microbial kinetics, substrate availability, and system hydraulics. Our results provide a clear quantitative framework for this balance, demonstrating that HRT ≥ 72 h is optimal for treating wastewater containing Diuron at concentrations up to 25 mg L^−1^, especially when combined with high carrier fill ratios.

The carrier filling ratio was identified as a statistically significant parameter influencing both COD and Diuron removal (*p* < 0.0001, ANOVA). As illustrated in Fig. 7 and 8, which depicts the interactive effects of HRT and carrier fill fraction on Diuron removal at low organic load (COD = 500 mg L^−1^, Diuron = 17.5 mg L^−1^), increasing the fill fraction from 30% to 70% significantly enhanced degradation efficiency. At HRT = 72 h, Diuron removal increased from 86.1% at 30% fill to 97.88% at 70% fill (Run 5). The three-dimensional response surface indicates that Diuron removal efficiencies exceeding 95% are attained only when the hydraulic retention time is greater than 60 h and the carrier filling ratio is maintained at or above 50%. This behavior is consistent with the established understanding that higher carrier fill fractions increase the available specific surface area for microbial attachment, thereby promoting biomass retention and improving mass transfer between the target pollutant and active degrading microorganisms. Moreover, the high Adequate Precision value of the model (23.24) reflects a strong signal-to-noise ratio, supporting the reliability of the predicted response surface for process optimization. The synergy between long HRT and high biofilm surface area was evident. The best performance (97.88% Diuron, 92.91% COD) occurred at 72 h + 70%, while the worst (49.67% Diuron, 42.7% COD) was observed at 24 h + 30% filling + COD = 1500 mg L^−1^ (Run 16). The carrier filling ratio emerged as a critical operational parameter, exerting a strong positive effect on both COD and Diuron removal (*p* < 0.0001). Our findings, which demonstrate that increasing the fill ratio from 30% to 70% can boost Diuron removal by over 11 percentage points (from 86.1% to 97.88%), are consistent with recent studies on biofilm reactors treating micropollutants. For instance, Liu *et al.* (2023) reported that increasing the carrier fill ratio from 40% to 60% in an MBBR enhanced the removal of sulfamethoxazole by 15%, attributing the improvement to increased biofilm surface area and reduced washout rates.^59^ Similarly, Brahmi *et al.* (2024) demonstrated that a fill ratio of 70% was optimal for achieving >90% removal of carbamazepine in a pilot-scale MBBR, highlighting the need for sufficient biomass loading to support cometabolic degradation pathways.^60^ The 3D response surface in Fig. 7 and 8 provides a powerful visual confirmation of this principle: the steep gradient of the surface along the carrier fill axis underscores its dominant influence over Diuron removal. Notably, even at suboptimal HRTs (*e.g.*, 48 h), Diuron removal improved from 75.48% at 30% fill to 89.1% at 70% fill (Run 18), indicating that while HRT is crucial, carrier surface area can partially compensate for shorter contact times. This finding has significant practical implications. In real-world applications where space or cost constraints limit reactor volume, operators can still achieve high removal efficiencies by operating at higher carrier fill ratios, provided that oxygen transfer and mixing remain adequate. However, our data also suggest a plateau effect beyond 70% fill, as no further significant gains were observed, implying saturation of enzymatic capacity or mass transfer limitations.^61^ Moreover, the interaction between carrier fill ratio and HRT (AB term, *p* = 0.0802) revealed that the benefit of higher fill ratios is more pronounced at longer HRTs. For example, at HRT = 72 h, increasing fill from 30% to 70% boosted Diuron removal by 11.78 percentage points, whereas at HRT = 24 h, the same increase yielded only a 3.6 percentage point gain (from 63.77% to 67.37%). This observation is corroborated by Rusten (2006), who noted that spherical HDPE carriers allow stable operation up to 70% fill without compromising hydrodynamics, but excessive filling risks impaired mass transfer.^62^ In summary, optimizing carrier fill ratio is not merely about maximizing biomass loading but about balancing microbial kinetics, mass transfer, and system hydraulics. Our results provide a clear quantitative framework for this balance, demonstrating that a fill ratio of 70% is optimal for treating wastewater containing Diuron at concentrations up to 25 mg L^−1^, especially when combined with long HRTs.

A significant decline in Diuron removal was observed with increasing influent concentration (*p* = 0.0366). At HRT = 72 h and 70% filling, removal dropped from 97.88% (10 mg L^−1^) to 88.7% (25 mg L^−1^) under COD = 500 mg L^−1^, indicating mild substrate inhibition. The observed behavior aligns with previous findings on biofilm-based treatment of pesticide-contaminated wastewater, where increased toxicant concentrations reduce enzymatic activity and impose diffusion limitations within biofilm layers.^47^ At 10 mg L^−1^, removal consistently exceeded 90% under optimized settings. At 25 mg L^−1^, efficiency declined slightly (to ∼90.5% under optimal conditions; 80–86% under suboptimal), indicating mild microbial inhibition. Nevertheless, the observed reduction (≤4%) suggests that the biofilm system maintains stable performance. The presence of elevated organic loading exerted a significant inhibitory effect on Diuron biodegradation, as confirmed by both statistical analysis (*p* = 0.0002) and visual inspection of Fig. 8. This phenomenon, known as substrate competition, occurs when heterotrophic microorganisms preferentially utilize readily biodegradable substrates (*e.g.*, glucose) over recalcitrant micropollutants like Diuron. Our findings align with recent studies that have documented similar trends for other xenobiotics. For instance, Liang *et al.* (2021) reported that increasing COD from 500 to 1500 mg L^−1^ reduced the removal of sulfamethoxazole by 12% in an MBBR, attributing the decline to competitive inhibition of cometabolic pathways.^63^ The 3D surface plot in Fig. 8 provides a powerful visualization of this interaction: the steep gradient along the organic load axis underscores its dominant influence over Diuron removal, particularly at shorter HRTs. Notably, even at suboptimal HRTs (*e.g.*, 24 h), increasing organic load from 500 to 1500 mg L^−1^ caused a 10.66 percentage point drop in Diuron removal, highlighting the sensitivity of micropollutant degradation to organic load variations. However, longer HRTs reduced the negative effect of high organic loads. For example, at HRT = 72 h, the reduction in Diuron removal due to increased organic load was only 7.5 percentage points (from 89.1% to 81.6%), compared to 10.66 percentage points at HRT = 24 h. This observation is corroborated by Chen *et al.* (2022), who noted that longer HRTs allow for the enrichment of specialized degraders capable of utilizing multiple substrates simultaneously, thereby reducing the competitive pressure on cometabolic pathways.^64^ Moreover, the interaction between organic load and HRT (AD term, *p* = 0.2686) revealed that the benefit of longer HRTs is more pronounced at higher organic loads. For example, at COD = 1500 mg L^−1^, increasing HRT from 24 to 72 h boosted Diuron removal by 31.34 percentage points (from 50.26% to 81.6%), whereas at COD = 500 mg L^−1^, the same increase yielded only a 28.18 percentage point gain (from 60.92% to 89.1%). When organic loading increased, Diuron removal declined unless sufficient contact time was provided, highlighting the sensitivity of the process to hydraulic conditions. While organic matter clearly interfered with removal, its effect diminished as retention time increased. At HRTs of 72 h or longer, system performance recovered to a large extent, even under high organic loads. This behavior has direct implications for the operation of MBBRs treating industrial wastewaters with fluctuating composition. The influence of organic load was examined at constant Diuron concentration (17.5 mg L^−1^) and a carrier fill of 50%. As shown in Fig. 7 and 8, which illustrates the interaction between HRT and organic load, increasing influent COD from 500 to 1500 mg L^−1^ resulted in a moderate decrease in Diuron degradation efficiency. At HRT = 72 h, removal declined from 89.1% at 500 mg per L COD to 81.6% at 1500 mg per L COD. Similar trends have been reported in biofilm reactors treating herbicides, where higher organic loads hinder pollutant-specific degradation by altering microbial community function and metabolic priorities.^44^ According to the 3D response surface, increasing HRT partially alleviates the adverse effect of high organic load; however, the overall pattern suggests competition between glucose and Diuron for microbial enzymatic machinery. Organic load was identified as a significant variable influencing Diuron removal by ANOVA analysis (*p* = 0.0002). The AC interaction term (HRT × Diuron concentration) was also statistically significant (*p* < 0.0001), demonstrating that organic load exerts a stronger effect at lower HRTs. This is illustrated at HRT = 24 h, where Diuron removal declined from 60.92% at 500 mg per L COD to 50.26% at 1500 mg per L COD. Similar trends have been documented in mixed-carbon biological systems, where easily degradable COD competes with xenobiotics for enzymatic activity, reducing cometabolic transformation of phenylurea herbicides such as Diuron.^19^

### 3.6. Microbial community structure

Gram staining showed that most cells in the mature biofilm were Gram-negative rods. To be sure the staining worked properly, the usual controls—*S. aureus* (positive) and *E. coli* (negative)—were checked first, and they behaved exactly as expected. The overall pattern resembles what other studies have described for systems treating phenylurea herbicides, where genera such as *Pseudomonas*, *Sphingomonas*, and *Burkholderia* often become dominant.^65,66^ Since we did not perform any molecular analysis, we had to rely mostly on the Gram-stain slides, supported by what is known from earlier reports. The stains showed mainly Gram-negative rods, which fits with the groups usually associated with enzymes needed to break down phenylurea herbicides. The cell shapes and staining behavior line up with common Diuron-degrading bacteria, though a definitive identification would require sequencing. During the experiment, the reactor slowly became better at removing Diuron, suggesting that the biofilm was settling into the conditions. A few irregular performance drops appeared while the system should have been stable; these were small and likely related to natural sloughing or short-term shifts inside the community. Still, the reactor held its overall performance, probably because several members of the biofilm can fill similar metabolic roles when conditions change.^67^ The Gram stain helped outline the general morphology of the biofilm, but it does not allow reliable identification of the organisms actually carrying out phenylurea degradation. Molecular approaches such as 16S rRNA gene sequencing would be required for that purpose, as they can detect genera like *Pseudomonas*, *Sphingomonas*, and *Comamonas*, which are often linked to the breakdown of Diuron and its metabolite 3,4-DCA.^68,69^ The strong presence of Gram-negative cells in our samples is consistent with established degradation pathways for substituted urea herbicides, where oxygenases and dehalogenases initiate transformation through *N*-demethylation and dechlorination steps, as documented in earlier biofilm studies.^47^ Throughout the experiment, the biofilm remained structurally stable and continued degrading the target compounds, even when the substrate loading was changed. Earlier studies have reported similar patterns, noting that mixed microbial communities can sustain cometabolic activity toward xenobiotics even when the amount of easily degradable COD shifts, which helps maintain stable removal in systems exposed to multiple substrates.^19^ Based on the behaviour observed, the biofilm seems to have developed into a robust and varied community able to handle both Diuron and organic matter reliably.

### 3.7. Comparative perspective and practical implications

Compared to conventional activated sludge (<50% phenylurea removal), the MBBR system demonstrated significantly higher removal efficiencies for both Diuron and COD. The reactor maintained stable performance even when operating conditions varied, indicating that the biofilm could tolerate shifts in influent quality. This behavior is useful in practice because agricultural and industrial wastewaters often show considerable variability in both organic loading and contaminant concentrations. Adsorption-based approaches can remove these compounds efficiently, but they come with practical drawbacks related to sorbent regeneration and disposal. Recent studies reinforce MBBR's competitiveness, reporting >85–90% removal of pharmaceuticals and personal care products under variable loading conditions.^41,53,70^ Existing advanced oxidation processes (AOPs), including ozonation and UV/persulfate systems, typically achieve 90–95% Diuron removal but are limited by high energy demand (0.3–0.8 kWh m^−3^) and formation of transformation byproducts, making MBBR a more economical pretreatment option before these tertiary processes.^23^ Given the persistence of phenylurea herbicides and their low regulatory limits (*e.g.*, 0.1 µg L^−1^ in EU drinking water standards), the effluent from the MBBR system can be further polished using granular activated carbon or ozonation to achieve full compliance. Hybrid MBBR–GAC trains have been reported to reduce residual pesticide concentrations by an additional 1–2 log units.^71^ The treatment system presented in this study can be applied as an effective pretreatment for agricultural effluents containing herbicides, especially where operational simplicity and low maintenance are priorities. The scalable nature of the process also makes it suitable for decentralized rural applications. Real agricultural wastewater often includes other organic pollutants—such as phenoxy acids, carbamates, or dye residues—that may influence which compounds are degraded preferentially. Biofilm systems often prioritize substrates with higher affinity for active enzymatic pathways, which can modify the removal profile for Diuron under multi-pollutant conditions.^47^ Furthermore, hydrodynamic conditions, including effluent velocity and shear forces, influence biofilm mass transfer and detachment rates; although these parameters were controlled in this study, future evaluations under variable flow regimes may offer additional insights for scale-up. From an operational perspective, MBBR systems incur relatively low costs due to limited aeration needs and long service life of carriers, while AOP-based tertiary polishing constitutes the major cost component, suggesting that the combined approach remains attractive for full-scale application.^72^ Overall, the findings provide valuable guidance for designing integrated treatment systems targeting herbicide-contaminated wastewater streams and highlight the complementary role of MBBR as a robust and cost-effective biological pretreatment technology.

## 4. Conclusion

This study shows that the lab-scale MBBR achieved effective removal of both Diuron and COD from synthetic wastewater. Under the applied operating conditions—an HRT of 71.7 h, a carrier fill of 52.6%, an organic load of 502.4 mg L^−1^, and an influent Diuron concentration of 10.13 mg L^−1^ the reactor removed 98.68% of Diuron and 93.4% of COD, values that exceed most previously reported results. Despite the high percentage removal, the Diuron concentration remaining in the effluent was still above the regulatory limit for direct discharge, indicating that the MBBR is more appropriate as a pre-treatment step prior to tertiary processes such as ozonation or activated carbon. The statistical results indicated that all of the tested variables—HRT, carrier fill, Diuron concentration, and organic load—had a measurable effect on how well the reactor performed. A lower organic load was particularly helpful, suggesting that competition for carbon affects co-metabolic degradation. Although the removal percentage was high, the final concentration of Diuron is still far from regulatory targets (*e.g.*, 212 µg L^−1^ compared with the EU limit of 0.1 µg L^−1^). In practical terms, this means the MBBR can reduce the herbicide load substantially, but it cannot replace a tertiary step if regulatory compliance is required. The overall trends observed in this work provide useful guidelines for designing MBBR systems for pesticide-containing wastewaters. The consistent performance across repeated runs also shows that the biofilm tolerated elevated Diuron levels without losing activity, which agrees with earlier work on mixed biofilms degrading phenylurea herbicides and intermediates such as 3,4-DCA. While significant reductions in both Diuron and COD were achieved, meeting very strict drinking-water-related limits generally requires an additional polishing treatment, particularly for phenylurea compounds with very low allowable concentrations. The behavior of the biofilm fits well with known degradation routes—mainly oxidative dealkylation and dechlorination—reported for similar substituted urea herbicides in biofilm-based systems.

### 4.1. Limitations and future directions

This work was carried out with synthetic wastewater, so tests with real agricultural or industrial effluents are still needed. Actual wastewater streams contain a mixture of pesticides, humic substances, and dissolved minerals that can alter degradation behavior and may require adjustments in process control to keep the reactor stable over long operation periods. Another limitation is that intermediate products—particularly 3,4-dichloroaniline (3,4-DCA)—were not measured, which restricts any conclusions about the extent of mineralization and potential toxicity. Tracking major transformation products such as 3,4-DCA by LC–MS/MS in later studies would provide clearer insight into degradation pathways and help establish a more complete mass balance. It would also be useful for future work to examine the microbial community through 16S rRNA gene sequencing to determine which organisms contribute to Diuron removal, as this information would strengthen both the mechanistic interpretation and the assessment of effluent-related risks. Hydrodynamic conditions—including shear forces, carrier motion, and superficial velocity—likely affect biofilm development and mass-transfer efficiency as well. Testing these factors under larger-scale flow conditions would offer practical guidance for designing reactors suitable for full-scale operation. Finally, longer operating tests are needed to evaluate the system's stability, its tolerance to shock loading, and its overall operating costs, especially when the process is combined with downstream tertiary treatment.

## Ethical statement

The study was approved by the Ethical Committee of Ardabil University of Medical Sciences, Iran (Code of ethics: IR.ARUMS.REC.1401.177).

## Conflicts of interest

No potential conflict of interest was reported by the authors.

## Data Availability

The data used to support the findings of this study are included within the article.
